# Non-autoimmune diabetes mellitus and the risk of virus infections: a systematic review and meta-analysis of case-control and cohort studies

**DOI:** 10.1038/s41598-021-88598-6

**Published:** 2021-04-26

**Authors:** Eric Lontchi-Yimagou, Charly Feutseu, Sebastien Kenmoe, Alexandra Lindsey Djomkam Zune, Solange Fai Kinyuy Ekali, Jean Louis Nguewa, Siméon Pierre Choukem, Jean Claude Mbanya, Jean Francois Gautier, Eugene Sobngwi

**Affiliations:** 1 Laboratory for Molecular Medicine and Metabolism, Biotechnology Center, University of Yaoundé 1, 3851 Yaoundé, Cameroon; 2 Department of Virology, Centre Pasteur of Cameroon, Yaoundé, Cameroon; 3 Department of Biochemistry and Molecular Biology, Faculty of Science, University of Buea, Buea, Cameroon; 4 Department of Internal Medicine and Specialties, Faculty of Medicine and Biomedical Sciences, University of Yaoundé 1, Yaoundé, Cameroon; 5 INSERM, Cordeliers Research Centre, Sorbonne Paris Cité, Université Paris Descartes, Université Paris Diderot, Paris, France; 6 Assistance Publique-Hôpitaux de Paris, Lariboisière Hospital, Department of Diabetes, Clinical Investigation Centre (CIC-9504), University Paris-Diderot, Paris, France; 7 Faculty of Medicine, University Paris-Diderot, Paris, France; 8 Department of Internal Medicine and Specialties, Faculty of Medicine and Pharmaceutical Sciences, University of Dschang, Dschang, Cameroon; 9 National Obesity Centre, Yaoundé Central Hospital, Yaoundé, Cameroon

**Keywords:** Endocrinology, Endocrine system and metabolic diseases, Diabetes, Type 2 diabetes

## Abstract

A significant number of studies invoked diabetes as a risk factor for virus infections, but the issue remains controversial. We aimed to examine whether non-autoimmune diabetes mellitus enhances the risk of virus infections compared with the risk in healthy individuals without non-autoimmune diabetes mellitus. In this systematic review and meta-analysis, we assessed case-control and cohort studies on the association between non-autoimmune diabetes and viruses. We searched PubMed, Embase, Cochrane Database of Systematic Reviews, Cochrane Central Register of Controlled Trials, and Web of Science with no language restriction, to identify articles published until February 15, 2021. The main outcome assessment was the risk of virus infection in individuals with non-autoimmune diabetes. We used a random-effects model to pool individual studies and assessed heterogeneity (*I*^2^) using the χ2 test on Cochrane’s Q statistic. This study is registered with PROSPERO, number CRD42019134142. Out of 3136 articles identified, we included 68 articles (90 studies, as the number of virus and or diabetes phenotype varied between included articles). The summary OR between non-autoimmune diabetes and virus infections risk were, 10.8(95% CI: 10.3–11.4; 1-study) for SARS-CoV-2; 3.6(95%CI: 2.7–4.9, *I*^2^ = 91.7%; 43-studies) for HCV; 2.7(95% CI: 1.3–5.4, *I*^2^ = 89.9%, 8-studies;) for HHV8; 2.1(95% CI: 1.7–2.5; 1-study) for H1N1 virus; 1.6(95% CI: 1.2–2.13, *I*^2^ = 98.3%, 27-studies) for HBV; 1.5(95% CI: 1.1–2.0; 1-study) for HSV1; 3.5(95% CI: 0.6–18.3 , *I*^2^ = 83.9%, 5-studies) for CMV; 2.9(95% CI: 1–8.7, 1-study) for TTV; 2.6(95% CI: 0.7–9.1, 1-study) for Parvovirus B19; 0.7(95% CI: 0.3–1.5 , 1-study) for coxsackie B virus; and 0.2(95% CI: 0–6.2; 1-study) for HGV. Our findings suggest that, non-autoimmune diabetes is associated with increased susceptibility to viruses especially SARS-CoV-2, HCV, HHV8, H1N1 virus, HBV and HSV1. Thus, these viruses deserve more attention from diabetes health-care providers, researchers, policy makers, and stakeholders for improved detection, overall proper management, and efficient control of viruses in people with non-autoimmune diabetes.

## Introduction

Diabetes mellitus (DM) is one of the most chronic, costly and fast-growing health challenges. In 2019, 463 million adults were estimated to have DM, and this number is expected to rise to 700 million by 2045 worldwide^1^. There are three main categories of diabetes: type 1 diabetes (T1D), type 2 diabetes (T2D) and gestational diabetes (GDM). T1D results from the destruction of insulin-producing pancreatic islet beta cells and T2D is caused by insufficient insulin production from beta cells, coupled with impaired insulin action in target tissues such as muscle, adipose tissue, and liver (a condition termed insulin resistance)^2^. GDM is characterized by high blood glucose levels during pregnancy. The prevalence of T2D in particular has markedly increased over the last 50 years in parallel with obesity, accounting for 85–95% of all diagnosed individuals with diabetes. Epidemiological data predict an inexorable and unsustainable increase in global health expenditure attributable to T2D, so disease prevention should be given high priority.

For an effective prevention of T2D associated complications, it is necessary to study and understand the risk factors involved. Multifactorial disorder and several different mechanisms have been implicated in the progression of T2D. The likely etiology is a combination of factors, including age, genetic inheritance, environmental factors, lifestyle, as well as infections which constitute a risk factor for developing T2D^3–10^. There is growing evidence for an etiological interaction between infectious diseases and T2D, as well as for the bidirectional influence of clinical presentation, spread, and outcomes^10, 11^. Moreover, a recent investigation suggests an interaction between a virus and ketosis-prone diabetes (KPD)^12^**.**

Viral infections seem to be strongly associated with non-autoimmune diabetes as viruses and T2D may coexist in an individual^13, 14^. Several studies reported that viruses can promote the increased prevalence of T2D^15^; however, whether T2D can increase the prevalence of viral infection still need to be elucidated. The repercussions associated with their infectivity may trigger T2D complications such as hypoglycemia and ketoacidosis^16^. An understanding of the complex association between virus infection and non-autoimmune diabetes is necessary for the design and development of novel drug therapies and lifestyle guidelines aimed at the treatment and/or prevention of these life-threatening diseases.

In this study, we assessed whether non-autoimmune diabetes is associated with an increased risk of contracting viruses compared to individuals without non-autoimmune diabetes. Establishing such evidence and defining the most common viruses found in individuals with non-autoimmune diabetes may lead to new paths and concepts for developing novel and specific preventive action and pharmacological treatment approaches of non-autoimmune diabetes-associated complications due to viruses.

## Methods

### Search strategy and selection criteria

This systematic review and meta-analysis was conducted according to the Preferred Reporting Items for Systematic Reviews and Meta-Analyses (PRISMA) standard (S1 Table) ^17^. The protocol was registered in the PROSPERO International prospective register of systematic reviews (registration number: CRD42019134142).

#### Types of studies

We considered case-control and cohort studies.

#### Participants

There is no specific filter for population. Individuals with non-autoimmune diabetes and without non-autoimmune diabetes (controls) were included.

#### Exposure

The exposure considered in this study is non-autoimmune diabetes diagnosed by a physician, or diagnosed based on the measured fasting plasma glucose, oral glucose tolerance test according to WHO criteria, or self-reported based on A_1C_ haemoglobin.

#### Outcomes

The outcome was defined for any virus infection diagnosed with serological or molecular techniques.

### Exclusion criteria

Case reports and secondary analyses, animal studies or studies reporting autoimmune type 1 diabetes outcomes only was excluded. Furthermore, we excluded letters, case reports and case series, review articles, editorials, commentaries, and cross-sectional studies.

### Literature search

Systematic literature searches were conducted by a medical librarian at Albert Einstein College of Medicine in the electronic databases PubMed (1947 to February 15, 2021), Embase (1973 to February 15, 2021), Cochrane Database of Systematic Reviews, Cochrane Central Register of Controlled Trials (Ovid) (1991 to February 15, 2021), and Web of Science (1985 to February 15, 2021). For all databases, both controlled vocabulary and text word searches were performed, using the combination of terms “diabetes”, “diabetes mellitus”, “non-autoimmune diabetes mellitus”, “type 2 diabetes mellitus”, “type II diabetes”, “T2D”, “T2DM”, “type 2 DM”, “non-insulin-dependent diabetes”, “NIDDM”, “ketosis-prone diabetes”, “fulminant diabetes”, “virus diseases” and “viral infection” (for the full search strategy see Appendix 1). The search was performed without any geographical limitation but limited to human studies (Appendix 1**)**. Manual hand searches of references from retrieved articles, major journals in the field, and grey literature (e.g., abstracts from scientific proceedings) were also performed to identify any additional relevant articles possibly missed by online indexes.

Two investigators (ELY, CF) independently identified articles and sequentially screen their title and abstracts for eligibility. Then, full texts of articles deemed potentially eligible were retrieved. Further, these investigators independently assessed the eligibility for inclusion in the review based on the inclusion and exclusion criteria. Additional information from study authors was performed to resolve questions about eligibility. Any disagreements between the two investigators were resolved through discussion and by consulting a third review author (LD) if necessary.

### Data extraction and management

Three investigators (CF, LD and SFKE) independently extracted data following the methods outlined in the Cochrane Handbook for systematic reviews of interventions. All the extracted data were cross-checked by a fourth investigator (ELY). All the disagreements were resolved through consensus. Data was collected on the first author name, year of publication, study design (case-control or cohort studies), geographical location (population where the study was performed), ethnicity, period of the study, number of the viruses screened, type of virus infection, the diagnostic technique used for the virus detection, diabetes phenotype, sample size (individuals with and without non-autoimmune diabetes), number of subjects infected (individuals with and without non-autoimmune diabetes), age and the gender (S2 Table). Any disagreement between the three investigators was resolved through discussion and by consulting a fourth investigator (ELY) if necessary.

### Assessment of methodological quality and data reporting

Three investigators (CF, LD, and SFKE) independently assessed study methodological quality of the included studies. Furthermore, all assessments were independently reviewed by a fourth investigator (ELY) and the disagreements were resolved by a consensus. A score for quality and bias, modified from the Newcastle–Ottawa scale, was used to assess each study. The scale assesses three domains: selection (five points), comparability, (two points), and outcome (three points) for a total score of 10 points. Studies scoring 7–10, 3–6, and 0–3 points were identified as having a low, moderate and high risk of bias respectively (S3 Table). Score disagreements were resolved by consensus and a final agreed-upon rating was assigned to each study.

### Data analysis

Study-specific estimates were pooled through a DerSimonian and Laird random-effects meta-analysis model^18^. The strength of the association was measured with odd ratios with 95% confidence interval (95%CI). We conducted subgroup analyzes according to study design (case-control and cohort studies), ethnicity (African, European, American and Asian) and type of non-autoimmune diabetes (T2D, KPD and gestational diabetes). We examined the robustness of the results with sensitivity analyses including only studies with a low risk of bias. Heterogeneity was evaluated by the χ^2^ test on Cochrane’s Q statistic which is quantified by I^2^ values, assuming that I^2^ values of 25%, 50% and 75% represent low, medium and high heterogeneity respectively ^19, 20^. The value of H close to 1 is indicative of some homogeneity between studies. We detected the publication bias through a visual inspection of funnel graphics and the Egger test^21^ with p < 0.10 indicating significant publication bias. To take into account the publication bias identified in the global analyses, we performed adjusted analyses by the Trim-and-fill approach^22^. The p < 0.05 values were indicative of a significant difference. We performed all the above analyses with the R version 3.5.1 software^23^.

## Results

### Study selection and characteristics

Of 3136 articles identified, 2901 remained after the elimination of duplicates. After screening titles and abstracts, we found 2716 articles to be irrelevant and we excluded them. We assessed full texts of the remaining 185 papers for eligibility, of which 117 were excluded**.** Sixty-eight (68) articles were included in this review for a total of 90 studies analyzed as the number of studied viruses and or diabetes phenotypes varied in between each included article (Fig. S1).

### Meta-analysis of the association between non-autoimmune diabetes mellitus and type-specific virus

We included 90 eligible studies^10, 12, 24–89^ in the meta-analysis for the measurement of the association between non-autoimmune diabetes and type-specific virus infection. Among these 90 studies, 82 (91.1%) were case-control and 8 (8.9%) were cohort studies (Table 1). The studies represented 4 ethnic groups globally: Africans (23.3%), Americans (10.0%), Asians (47.8%), and Europeans (18.9%) (Table 1). Sixty-three (70%) studies had a low risk of bias and 30 (30%) had a moderate risk of bias in their methodological quality. None had a high risk of bias (Table 1).

**Table 1:** Characteristics of included studies.

Characteristics	Number (N)	Percentage (%)
**Risk of bias**		
Moderate	27	30
Low	63	70
Total	90	100
**Study design**		
Case-control	82	91.1
Cohort	8	8.9
Total	90	100
**Diagnostic techniques**		
Immunoassay	52	57.8
Molecular assay	12	13.3
Molecular assay + immunoassay	21	23.3
Unclear/Not reported	5	5.6
Total	90	100
**Type of DM**		
T2D	89	98.9
KPD	1	1.1
Total	90	100
**Virus type**		
CMV	5	5.6
SARS-CoV-2	1	1.1
Coxsackie B virus	1	1.1
H1N1 Virus	1	1.1
HBV	27	30.0
HCV	43	47.8
HGV	1	1.1
HHV8	8	8.9
HSV1	1	1.1
Parvovirus B19	1	1.1
TTV	1	1.1
Total	90	100
**Ethnicity**		
Africans	21	23.3
Americans	9	10.0
Asians	43	47.8
European	17	18.9
Total	90	100

A total of 11 viruses were reported: HCV (43 studies)^10, 24–60, 82, 84, 86, 88, 89^, HBV (27 studies)^10, 30, 35, 36, 38, 41, 44, 48, 52, 53, 56, 59, 60, 66–74, 82, 84, 85^, HHV8 (8 studies)^12, 61–65, 81^, CMV (5 studies)^76–80^, SARS-CoV-2 (1 study)^83^, H1N1 virus (1 study)^87^, HSV1 (1 study)^75^, TTV (1 study)^37^, Parvovirus B19 (1 study)^79^, coxsackie B (1 study)^79^ and HGV (1 study)^37^ (Table 1 ).

**Figure 1:**
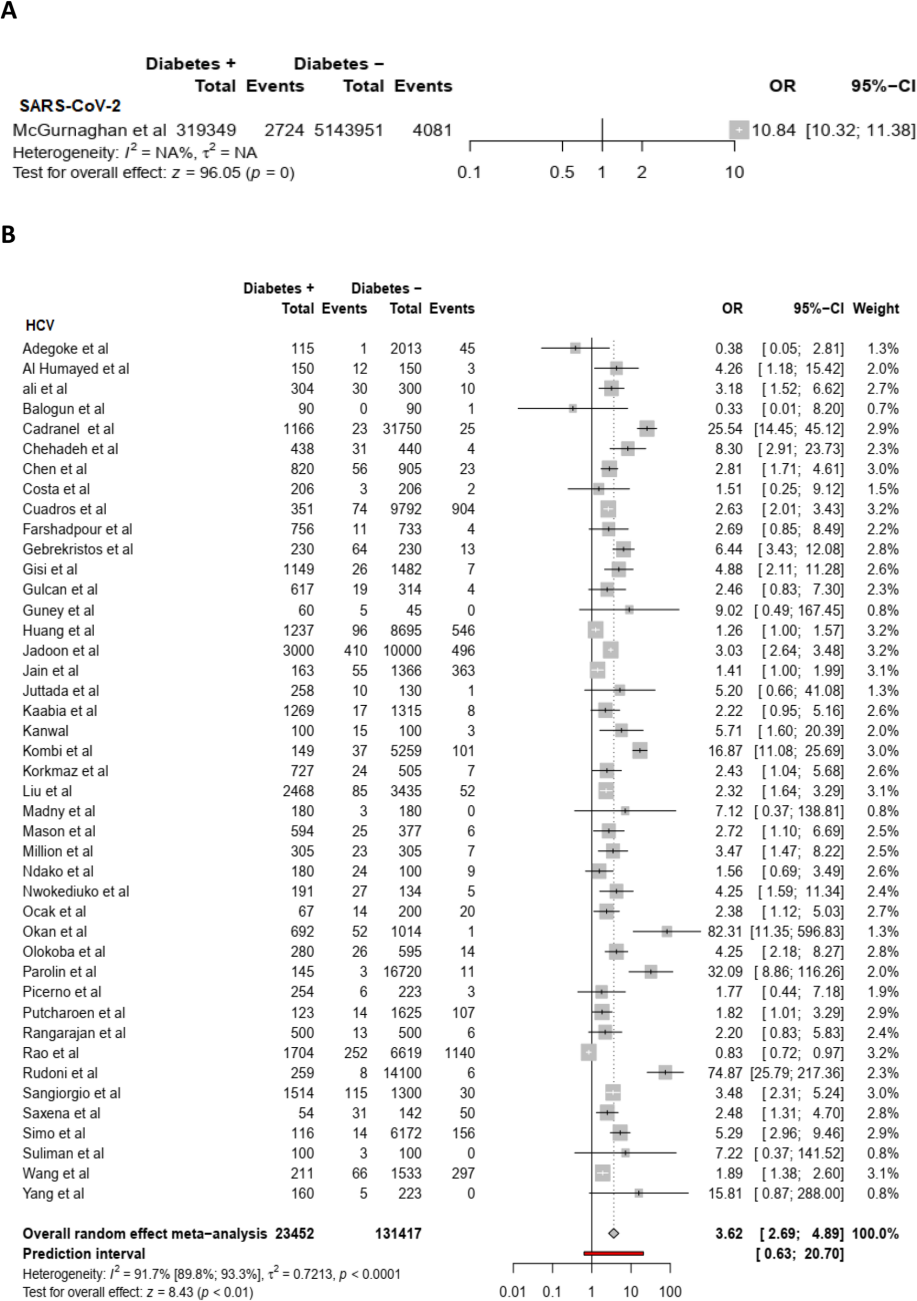
Meta-analysis of the association between SARS-CoV-2 (**A**), HCV (**B**) and non-autoimmune diabetes mellitus.

**Figure 2:**
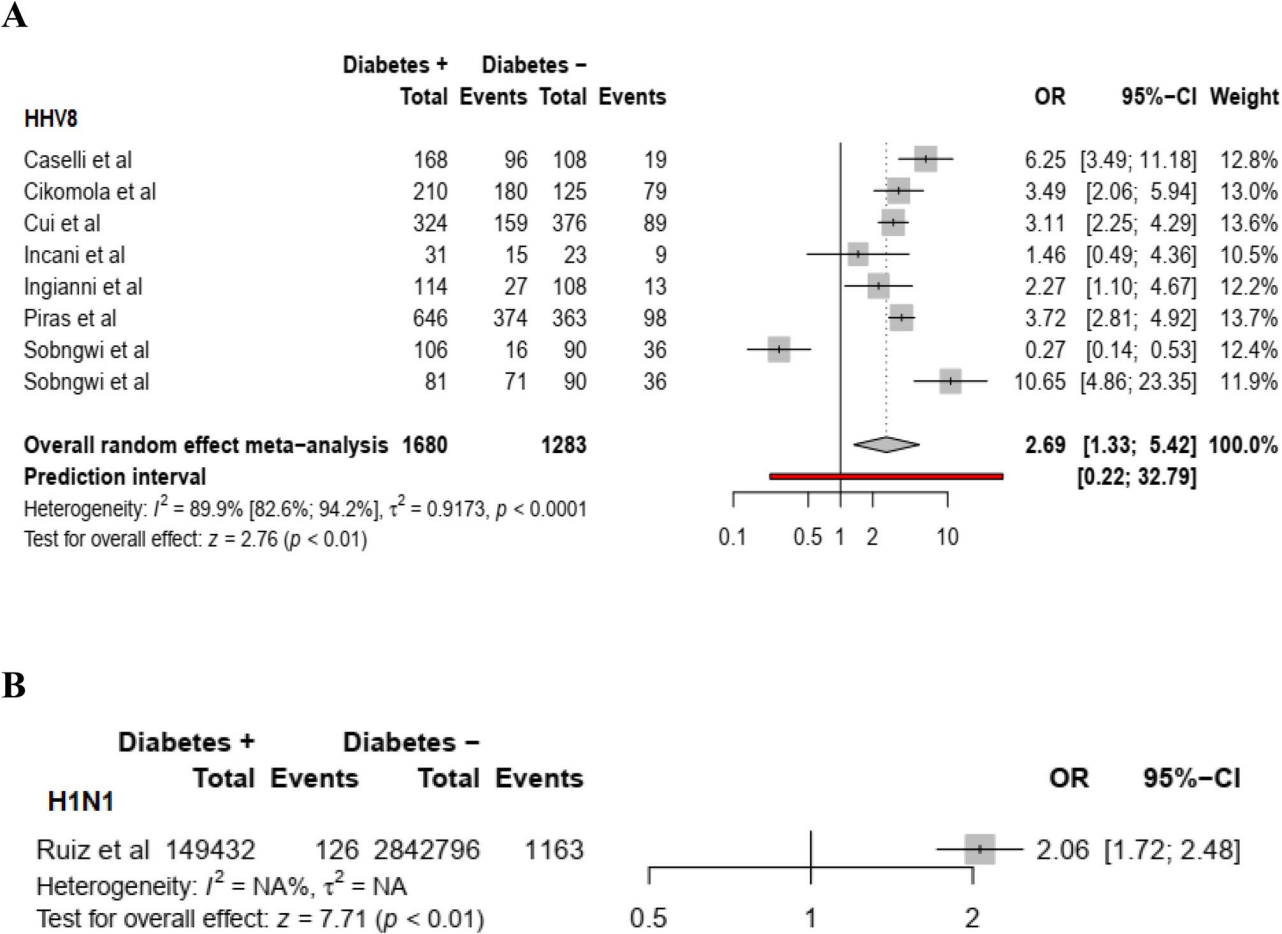
Meta-analysis of the association between HHV8 (**A**), H1N1 virus (**B**) and non-autoimmune diabetes mellitus.

**Figure 3:**
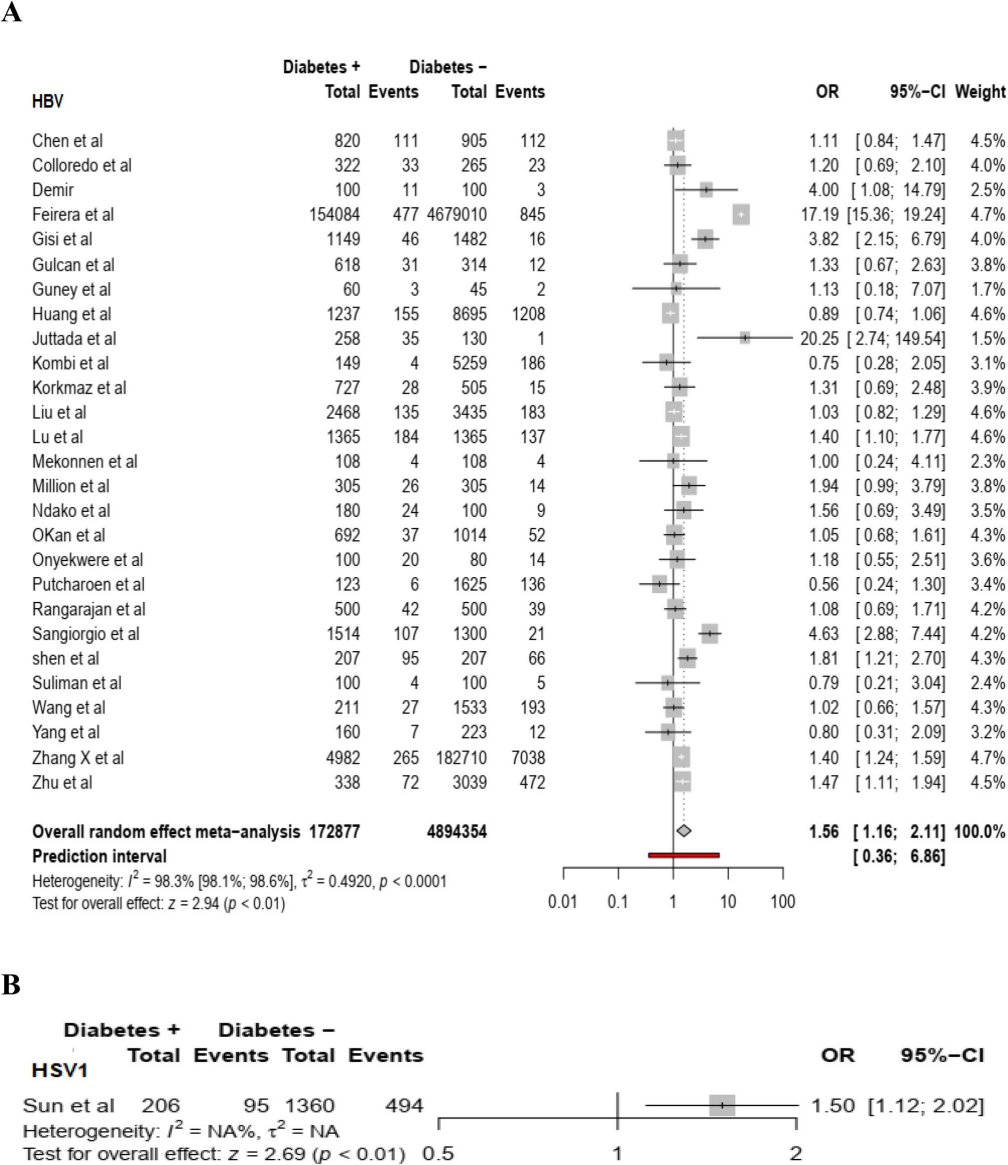
Meta-analysis of the association between HBV (**A**), HSV1 (**B**) and non-autoimmune diabetes mellitus.

Based on random effect model, individuals with non-autoimmune diabetes were at a higher risk of acquiring SARS-CoV-2 (summary OR = 10.8 (95% CI: 10.3–11.4), HCV (summary OR = 3.6 (95% CI:2.5–4.9, *I*^2^ = 0.91.7%), HHV8 (summary OR = 2.7 (95%CI:1.3–5.4; *I*^*2*^ = 89.9), H1N1 virus (summary OR = 2.1(95% CI: 1.7–2.5), HBV (summary OR = 1.6 (95% CI: 1.2–2.1, *I*^*2*^ = 98.3%), and HSV1 (summary OR = 1.5 95% CI: 1.12–2.02) infection compared to individuals without non-autoimmune diabetes [(Fig. 1A,B for COVID-19 and HCV), (Fig. 2A,B for HHV8 and H1N1), (Fig. 3A,B for HBV and HSV1) respectively]. The funnel plot suggests publication bias for HCV infection (S2 Fig.) whereas there was no publication bias for HHV8 (S3 Fig.) and HBV (S4 Fig.) infection. These results were confirmed by the Egger test (Table 2). Furthermore, the OR in studies with a low risk of bias was not different from the overall OR. When the Trim and Fill method was performed, the OR for HCV infection was attenuated but remained statistically significant (OR = 1.9 (95% CI: 1.3–2.8). Substantial heterogeneity was present overall and within all subgroups (Table 2).

**Table 2:** Summary statistic of the association between non-autoimmune diabetes mellitus and type-specific virus.

Virus	OR (95%CI)	95% Prediction interval	N Studies	N Cases	N Controls	H (95%CI)	I^2^ (95%CI)	P Heterogeneity	P Egger test
**SARS-COV-2**									
Overall	10.8 [10.3–11.4]	NA	1	319,349	5,143,951	NA	NA	1	NA
Low risk of bias	10.8 [10.3–11.4]	NA	1	319,349	5,143,951	NA	NA	1	NA
**HCV**									
Overall	3.6 [2.7–4.9]	[0.6–20.7]	43	23,452	131,417	3.5 [3.1–3.9]	91.7 [89.8–93.3]	< 0.001	0.001
Low risk of bias	4.5 [3–6.6]	[0.6–32.4]	30	20,362	112,544	4.1 [3.7–4.6]	94.1 [92.5–95.3]	< 0.001	0.001
Trim-and-fill adjusted analysis	1.9 [1.3–2.8]	[0.1–30.5]	59	NA	NA	3.8 [3.5–4.1]	93.2 [91.9–94.3]	< 0.001	0.645
**HHV8**									
Overall	2.7 [1.3–5.4]	[0.2–32.8]	8	1680	1283	3.2 [2.4–4.2]	89.9 [82.6–94.2]	< 0.001	0.46
Low risk of bias	2.8 [1.1–6.9]	[0.1–74.6]	6	1439	1135	3.7 [2.7–4.9]	92.6 [86.6–95.9]	< 0.001	0.564
**H1N1 virus**									
Overall	2.1 [1.7–2.5]	NA	1	149,432	2,842,796	NA	NA	1	NA
Low risk of bias	2.1 [1.7–2.5]	NA	1	149,432	2,842,796	NA	NA	1	NA
**HBV**									
Overall	1.6 [1.2–2.1]	[0.4–6.9]	27	172,877	4,894,354	7.8 [7.2–8.4]	98.3 [98.1–98.6]	< 0.001	0.887
Low risk of bias	1.7 [1.2–2.4]	[0.3–8.4]	20	170,684	4,889,141	9 [8.2–9.8]	98.8 [98.5–99]	< 0.001	0.749
**HSV1**									
Overall	1.5 [1.1–2]	NA	1	206	1360	NA	NA	1	NA
**CMV**									
Overall	3.3 [0.6–18.3]	[0–1524.7]	5	208	179	2.5 [1.7–3.7]	83.9 [63.7–92.9]	< 0.001	0.286
Low risk of bias	6.4 [0.5–77.7]	[0.41–48.4]	3	81	105	3 [1.8–5]	89.1 [70.3–96]	< 0.001	0.468
**TTV**									
Overall	2.9 [1–8.7]	NA	1	60	45	NA	NA	1	NA
Low risk of bias	2.9 [1–8.7]	NA	1	60	45	NA	NA	1	NA
**Parvovirus B19**									
Overall	2.6 [0.7–9.1]	NA	1	83	30	NA	NA	1	NA
**Coxsackie B virus**									
Overall	0.7 [0.3–1.5]	NA	1	83	30	NA	NA	1	NA
**HGV**									
Overall	0.2 [0–6.2]	NA	1	60	45	NA	NA	1	NA
Low risk of bias	0.2 [0–6.2]	NA	1	60	45	NA	NA	1	NA

*CMV* Cytomegalovirus; *SARS-CoV-2* Severe Acute Respiratory Syndrome Coronavirus 2; *HBV* Hepatitis B Virus; *HCV* Hepatitis C Virus; *HGV* Hepatitis G Virus; *HHV8* Human Herpes Virus 8; *HSV1* Herpes Simplex Virus 1; *TTV* Transfusion Transmitted Virus.

**Figure 4:**
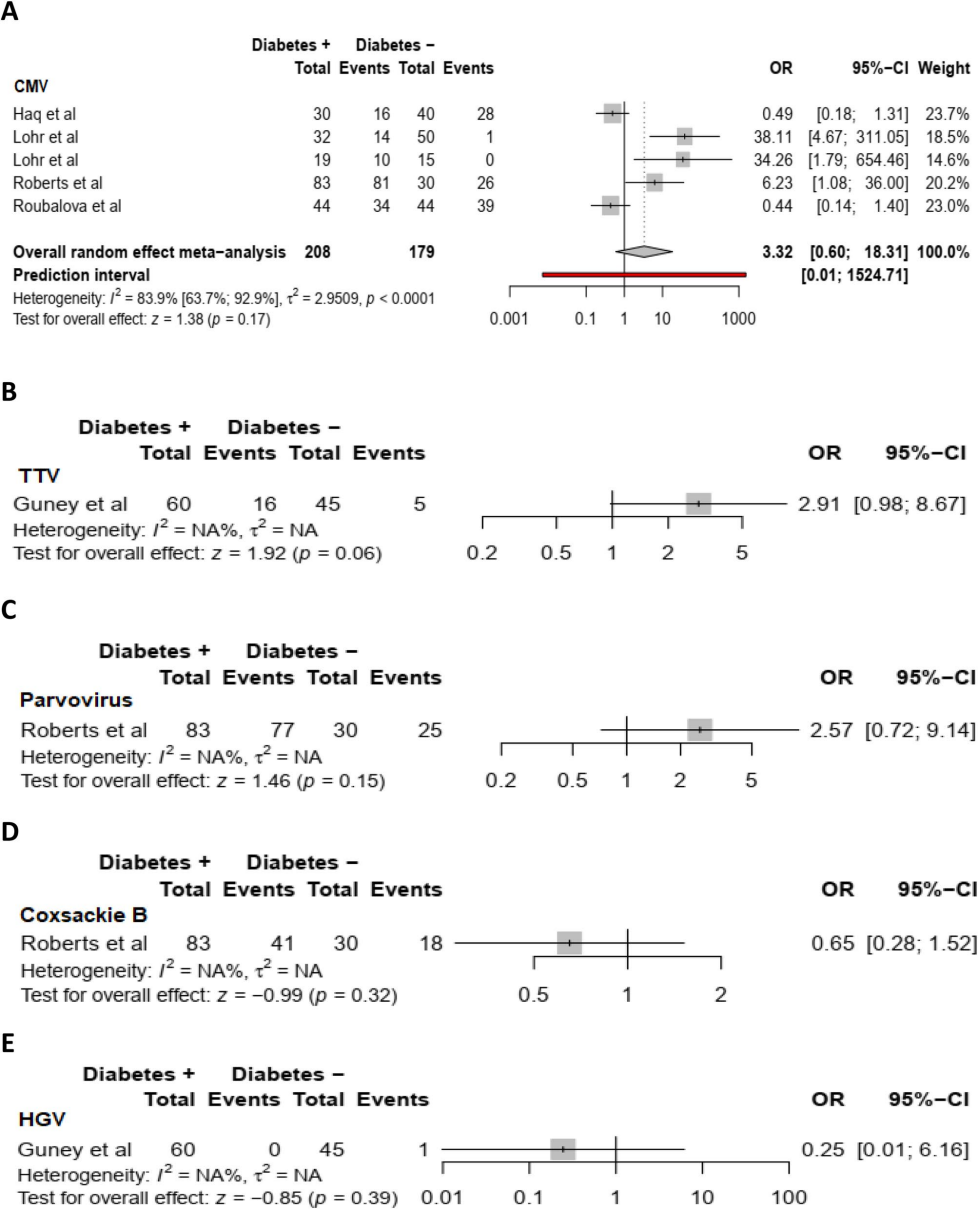
Meta-analysis of the association between CMV (**A**), TTV (**B**), Parvovirus B19 (**C**), Coxsackie B virus (**D**), HGV (**E**) and non-autoimmune diabetes mellitus.

We found the risk for non-autoimmune diabetes individuals to acquire CMV (summary OR = 3.3 (95%CI:0.6–18.3, *I*^*2*^ = 83.9%) (Fig. 4A), TTV (summary OR = 2.9(95% CI: 0.98–8.67) (Fig. 4B), parvovirus B19 (summary OR = 2.6(95% CI: 0.72–9.14) (Fig. 4C), coxsackie B virus (summary *OR* = 0.7 95% CI: 0.3–1.5) (Fig. 4D) and HGV (summary OR: 0.2 95% CI: 0–6.2) (Fig. 4E) infections to be non-statistically significant compared to healthy individuals without non-autoimmune diabetes.

For CMV infection, the funnel plot did not suggest publication bias (S5 Fig.), and this result was confirmed by the Egger test. The OR in studies with low risk of bias (summary OR = 6.495% CI: 0.5–77.7) was different from the overall OR and substantial heterogeneity was present overall and within all subgroups (Table 2).

### Subgroup analysis

With respect to ethnicity, the risk of HCV, HHV8 and HBV infection in individuals with non-autoimmune diabetes was higher in the European population than in other ethnic groups. According to type of non-autoimmune diabetes, the HHV8 infection risk was higher in KPD individuals (summary OR = 10.7 [95% CI: 4.9–23.3]) compared to T2D individuals (summary OR = 2.2 [95% CI: 1.1—4.5]; *I*^2^ = 90%). For diagnostic techniques used to detect virus infection, the risk of CMV and HCV infection in individuals with non-autoimmune diabetes was statistically different according to diagnostic techniques (immunoassay alone, molecular assay alone or molecular assay and immunoassay) (S4 Table).

## Discussion

This systematic review and meta-analysis aimed to examine the association between non-autoimmune diabetes and the risk of acquiring virus infection compared to the general populations without non-autoimmune diabetes. In this study, we included 90 studies which assessed and compared the risk of virus infection in individuals with or without non-autoimmune diabetes. The results of this meta-analysis indicate an increased risk of SARS-CoV-2, HCV, HHV8, H1N1 virus, HBV, and HSV1 infection, which is statistically significant amongst individuals with non-autoimmune diabetes when compared to individuals without non-autoimmune diabetes.

Strikingly, we found in this study a 3.6-fold higher risk for individuals with non-autoimmune diabetes to acquire HCV infection. These results are consistent with a meta-analysis performed by Guo et al. in 2013 who reported an approximated 3.5-fold increase in HCV infection risk in individuals with T2D^90^. Indeed, HCV infection seems to be strongly associated with non-autoimmune diabetes. However, the direction of this association is not fully elucidated. A meta-analysis performed in 2012 by Naing et al. reported an excessive T2D risk in HCV-infected individuals^91^. More specifically, they found an about 1.7-fold increase in T2D risk in HCV infected individuals compared with non-infected individuals^91^. White et al. in 2009 in a meta-analysis demonstrated an approximately 1.7-fold significant increase in non-autoimmune diabetes risk with HCV infection^15^. Fabiani et al. in 2018 showed that HCV infection is associated with an increased risk of T2DM independently from the severity of the associated liver disease, in hepatitis C virus infection (CHC) and cirrhotic HCV individuals. As expected T2DM risk is higher in cirrhotic HCV patients than CHC, and the prevalence of HCV infection in T2DM patients is higher than in individuals without non-autoimmune diabetes ^92^**.** Thus, the question that arises is: does HCV precede non-autoimmune diabetes or vice-versa? Lim et al. in 2019 demonstrated that chronic hepatitis C patients have profound subcutaneous adipose tissue insulin resistance in comparison with BMI-matched controls and viral eradication improves global, hepatic and adipose tissue insulin sensitivity^93^, suggesting that HCV probably precedes non-autoimmune diabetes.

In the current meta-analysis, a 1.6- fold increase in HBV infection risk in individuals with non-autoimmune diabetes was reported. Cai et al. performed a meta-analysis in 2015 and found that the OR for the prevalence of diabetes mellitus was 1.33 (95% CI, 1.09–1.62; p = 0.005) between the individuals with and without HBV infection^94^. Two recent meta-analyses also reported that the summary OR of the risk of T2DM for hepatitis B cirrhosis patients was 1.99 (95% CI, 1.08–3.65) and 1.76 (95% CI: 1.44–2.14) when compared with the non-HBV individuals^95, 96^. The lower risk of HBV infection in individuals with non-autoimmune diabetes mellitus compared to HCV could be explained partly by the fact that Hepatitis B has been controlled in most countries, through active HBV vaccination programs. Also, the occurrence of chronic HBV and its complications in these countries is very low.

We report in this meta-analysis a 10.8-fold, 2.7-fold, 2.1-fold and 1.5-fold increase in SARS-CoV-2, HHV8, H1N1 virus and HSV1 infection risk respectively when individuals with and without non-autoimmune diabetes were compared. However, the association between non-autoimmune diabetes and these viruses was limited by the lower number of studies eligible for our study and/or the small sample size of the included studies. Though little is known about non-autoimmune diabetes been a risk factor for SARS-CoV-2infection, several studies have indicated COVID-19 severity in patients with diabetes through, increase in ACE-2 and furin expression, impaired T-cell function and increased interleukin-6^97^. Similarly, about a decade ago, diabetes mellitus has been associated with increased severity and hospitalization with H1N1 virus infection ^98, 99^. HHV8 infection can induce an inflammatory state through the activation of reactive oxygen species and the production of acute-phase proteins which can have a fundamental role in metabolic modification and can lead to the onset of KPD^100^. It may also influence the pathogenesis of non-autoimmune diabetes either by cytokine secretion or by direct infection of the pancreatic β cell, or both^12^. Nguewa et al. found that symptomatic HHV-8 infection does not appear to be associated with decreased insulin sensitivity in individuals with non-autoimmune diabetes ^101^. However, Lontchi-Yimagou et al. in 2018 found that the positivity of HHV8 DNA in individuals with non-autoimmune diabetes was associated with low insulin secretion ^102^.

In this meta-analysis, we found a low and statistically not significant risk for individuals with non-autoimmune diabetes to acquire TTV, Parvovirus B19, Coxsackie B and HGV infection. Very few studies in the literature assessed the association between non-autoimmune diabetes and these viruses. Nevertheless, some studies report an association between viruses, (in particular enteroviruses) and type 1 diabetes ^103–106^. In a meta-analysis, Yeung et al., in 2011, reported a significant association between enteroviruses and type 1 diabetes-related autoimmunity and clinical type 1 diabetes ^103^.

In subgroup analysis according to geographic area, we reported a high risk of HCV, HBV and HHV8 in the European population with non-autoimmune diabetes mellitus. However, this disparity can be due to the lack of epidemiological studies assessing the risk of these virus infections in individuals with non-autoimmune diabetes in other areas, mainly in the African region where the prevalence of non-autoimmune diabetes and virus infection increased drastically during the last decade. For this reason, it is urgent to perform robust epidemiological studies in this region to fill this gap and develop approaches to break the bridge between non-communicable diseases and infectious diseases.

This study provides an insight into the question of whether individuals with non-autoimmune diabetes have an increased susceptibility to virus infections. Additionally, the results of this meta-analysis may have important clinical implications; given the demonstrated increased risk of some virus infection in individuals with non-autoimmune diabetes, it would be important to screen for those virus infections in individuals with non-autoimmune diabetes.

However, the main limitation of our meta-analysis was the substantial heterogeneity among studies which could be explained partially by the variation in the sample size of the primary studies. Moreover, differentiating T1D from T2D at mid-adulthood is a challenge raising the possible misdiagnosis of T1D in the included studies^107^. Indeed, Atkinson et al. in 2014, reported that about 50% of T1D are misdiagnosed as T2D^108^. Furthermore, due to the few numbers of some studies assessing the association between virus type infection and non-autoimmune diabetes, we were unable to perform subgroup analysis for those studies. Finally, the heterogeneity in the results could also be explained by the use of different techniques (including, ELISA, PCR, immunofluorescence, RT-qPCR, rapid immunochromatography) to detect the viruses in the included studies.

Viruses have been appealing to explain the increasing prevalence of diabetes, seasonal variation in onset ^109^**,** and enhanced susceptibility of trans-migratory populations ^110^**.** Indeed, viruses in the context of genetic associations may trigger the development of diabetes following several mechanisms including: direct destruction of pancreatic beta cells, increasing inflammation, increase insulin requirement, increasing insulin resistance, molecular mimicry, increased processing and presentation of autoantigens during infection. Some viruses may directly induce diabetes, while others have an indirect action on the development of diabetes.

The potential mechanisms of non-autoimmune diabetes favoring viral infection include; a hyperglycemic environment that increases the virulence of some pathogens; lower production of interleukins in response to infection; reduced chemotaxis and phagocytic activity, immobilization of polymorphonuclear leukocytes; glycosuria, gastrointestinal and urinary dysmotility. Further studies are required to determine on one hand, whether the risk of virus infection could be prevented or reversed with the improvement of glycemic control in individuals with non-autoimmune diabetes mellitus and on the other hand, whether the eradication of virus infection with appropriate therapy can reduce or abolish the onset of diabetes in these individuals.

## Conclusion

This study suggests that non-autoimmune diabetes mellitus is associated with increased susceptibility to viruses especially SARS-CoV-2, HCV, HHV8, H1N1, HBV and HSV1. Thus, these virus infections deserve more attention from diabetes health-care providers, researchers, policy makers, and stakeholders for improved detection, overall proper management, and efficient control of viruses in people with non-autoimmune diabetes mellitus. This systematic review and meta-analysis also suggest the need for more extensive studies that include larger populations. There is sufficient empirical evidence to justify greater research emphasis on the interaction between viruses and non-autoimmune diabetes.

## Data availability

Data are available as supplementary material.

## Supplementary Information

Supplementary Information.
