# Supplementary material for: Non-autoimmune diabetes mellitus and the risk of virus infections: a systematic review and meta-analysis of case-control and cohort studies
Source: Sci Rep. 2021 Apr 26;11:8968. doi: 10.1038/s41598-021-88598-6 (PMC8076178; doi:10.1038/s41598-021-88598-6)
Supplement: Supplementary file 1 — Supplementary Information. [file 41598_2021_88598_MOESM1_ESM.docx]

# Non-autoimmune Diabetes Mellitus and the Risk of Virus Infections: A Systematic Review and Meta-analysis of case-control and cohort studies

Eric Lontchi-Yimagou, Charly Feutseu, Sebastien Kenmoe, Alexandra Lindsey Djomkam Zune, Solange Fai Kinyuy Ekali, Jean Louis Nguewa, Simeon Pierre Choukem, Jean Claude Mbanya, Jean Francois Gautier, Eugene Sobngwi

**Appendix**

1. **Database Search Strategies**

**PubMed**

((((((((((((fulminant diabetes[Title/Abstract]) OR ((((((NIDDM[Title/Abstract]) OR non-autoimmune diabetes[Title/Abstract]) OR ketosis-prone diabetes[Title/Abstract]) OR non-insulin dependent diabetes[Title/Abstract]) OR type 2 diabetes[Title/Abstract]) OR T2DM[Title/Abstract]))) OR non-autoimmune diabetes type 1) OR diabetes mellitus, type 2[MeSH Terms])) AND (((virus diseases[MeSH Terms]) OR viral infection*[Title/Abstract]) OR virus*[Title/Abstract]))) AND ((((((((((((cohort studies[MeSH Terms]) OR cross-sectional studies[MeSH Terms]) OR case-control studies[MeSH Terms]) OR randomized controlled trial[Publication Type])) OR (((((control groups[Title/Abstract]) OR ((case*[Title/Abstract]) AND comparison[Title/Abstract])) OR ((cases[Title/Abstract]) AND controlled[Title/Abstract])) OR ((cases[Title/Abstract]) AND controls[Title/Abstract])) OR ((case[Title/Abstract]) AND control[Title/Abstract]))) OR (((clinical trial[Publication Type]) OR "clinical trials as topic"[MeSH Terms]) OR "double-blind method"[MeSH Terms])) OR ((((randomized[Title/Abstract]) OR randomised[Title/Abstract])) AND ((trial[Title/Abstract]) OR trials[Title/Abstract]))) OR ((control*[Title/Abstract]) AND ((((randomized[Title/Abstract]) OR randomised[Title/Abstract])) AND ((trial[Title/Abstract]) OR trials[Title/Abstract])))) OR ((((blind[Title/Abstract]) OR mask*[Title/Abstract])) AND ((((((single[Title/Abstract]) OR double[Title/Abstract]) OR doubled[Title/Abstract]) OR triple[Title/Abstract]) OR tripled[Title/Abstract]) OR treble[Title/Abstract])))) OR ((systematic review[Title/Abstract]) OR meta-analysis[Title/Abstract])))) AND english[Language])) NOT "Case Reports"[Publication Type]

**Embase**

('non insulin dependent diabetes mellitus'/exp OR 'non-insulin dependent diabetes':ab,ti OR 'ketosis prone diabetes':ab,ti OR 'non-autoimmune diabetes':ab,ti OR 'nonautoimmune diabetes':ab,ti OR 'niddm':ab,ti OR 't2dm':ab,ti OR 't2d':ab,ti) AND ('viral infection':ab,ti OR (virus AND diseases:ab,ti) OR 'virus diseases':ab,ti OR 'dna virus':ab,ti OR 'rna virus':ab,ti) AND [humans]/lim AND [english]/lim AND [embase]/lim NOT ([embase]/lim AND [medline]/lim) NOT 'case report'/exp

**Web of Science**

TS=(fulminant diabetes OR NIDDM OR non-autoimmune diabetes OR ketosis-prone diabetes OR non-insulin dependent diabetes OR type 2 diabetes OR T2DM OR non-autoimmune diabetes type 1 OR diabetes mellitus, type 2) AND TS=(virus diseases OR viral infection* OR virus*)

**Cochrane Database of Systematic Reviews & Cochrane Controlled Trials Register (Ovid)**

(fulminant diabetes OR NIDDM OR non-autoimmune diabetes OR ketosis-prone diabetes OR non-insulin dependent diabetes OR type 2 diabetes OR T2DM OR non-autoimmune diabetes type 1) AND (virus diseases OR viral infection OR virus*)

1. **Supplementary tables**
   1. Supplementary table 1. PRISMA 2009 checklist

| **Section/topic** | **#** | **Checklist item** | **Reported on page #** |
| --- | --- | --- | --- |
| **TITLE** | | |  |
| Title | 1 | Identify the report as a systematic review, meta-analysis, or both. | 1 |
| **ABSTRACT** | | |  |
| Structured summary | 2 | Provide a structured summary including, as applicable: background; objectives; data sources; study eligibility criteria, participants, and interventions; study appraisal and synthesis methods; results; limitations; conclusions and implications of key findings; systematic review registration number. | 2 |
| **INTRODUCTION** | | |  |
| Rationale | 3 | Describe the rationale for the review in the context of what is already known. | 4-5 |
| Objectives | 4 | Provide an explicit statement of questions being addressed with reference to participants, interventions, comparisons, outcomes, and study design (PICOS). | 4-5 |
| **METHODS** | | |  |
| Protocol and registration | 5 | Indicate if a review protocol exists, if and where it can be accessed (e.g., Web address), and, if available, provide registration information including registration number. | 6 |
| Eligibility criteria | 6 | Specify study characteristics (e.g., PICOS, length of follow-up) and report characteristics (e.g., years considered, language, publication status) used as criteria for eligibility, giving rationale. | 6 |
| Information sources | 7 | Describe all information sources (e.g., databases with dates of coverage, contact with study authors to identify additional studies) in the search and date last searched. | 6-7 |
| Search | 8 | Present full electronic search strategy for at least one database, including any limits used, such that it could be repeated. | Appendix |
| Study selection | 9 | State the process for selecting studies (i.e., screening, eligibility, included in systematic review, and, if applicable, included in the meta-analysis). | 6 |
| Data collection process | 10 | Describe method of data extraction from reports (e.g., piloted forms, independently, in duplicate) and any processes for obtaining and confirming data from investigators. | 6 |
| Data items | 11 | List and define all variables for which data were sought (e.g., PICOS, funding sources) and any assumptions and simplifications made. | 6 |
| Risk of bias in individual studies | 12 | Describe methods used for assessing risk of bias of individual studies (including specification of whether this was done at the study or outcome level), and how this information is to be used in any data synthesis. | 6 |
| Summary measures | 13 | State the principal summary measures (e.g., risk ratio, difference in means). | 6 |
| Synthesis of results | 14 | Describe the methods of handling data and combining results of studies, if done, including measures of consistency (e.g., I^2^) for each meta-analysis. | 6-7 |

| **Section/topic** | **#** | **Checklist item** | **Reported on page #** |
| --- | --- | --- | --- |
| Risk of bias across studies | 15 | Specify any assessment of risk of bias that may affect the cumulative evidence (e.g., publication bias, selective reporting within studies). | 6-7 |
| Additional analyses | 16 | Describe methods of additional analyses (e.g., sensitivity or subgroup analyses, meta-regression), if done, indicating which were pre-specified. | 6-7 |
| **RESULTS** | | |  |
| Study selection | 17 | Give numbers of studies screened, assessed for eligibility, and included in the review, with reasons for exclusions at each stage, ideally with a flow diagram. | 7-10 |
| Study characteristics | 18 | For each study, present characteristics for which data were extracted (e.g., study size, PICOS, follow-up period) and provide the citations. | 7-10 |
| Risk of bias within studies | 19 | Present data on risk of bias of each study and, if available, any outcome level assessment (see item 12). | 7-10 |
| Results of individual studies | 20 | For all outcomes considered (benefits or harms), present, for each study: (a) simple summary data for each intervention group (b) effect estimates and confidence intervals, ideally with a forest plot. | 7-10 |
| Synthesis of results | 21 | Present results of each meta-analysis done, including confidence intervals and measures of consistency. | 7-10 |
| Risk of bias across studies | 22 | Present results of any assessment of risk of bias across studies (see Item 15). | 7-10 |
| Additional analysis | 23 | Give results of additional analyses, if done (e.g., sensitivity or subgroup analyses, meta-regression [see Item 16]). | 7-10 |
| **DISCUSSION** | | | 10-12 |
| Summary of evidence | 24 | Summarize the main findings including the strength of evidence for each main outcome; consider their relevance to key groups (e.g., healthcare providers, users, and policy makers). | 10-12 |
| Limitations | 25 | Discuss limitations at study and outcome level (e.g., risk of bias), and at review-level (e.g., incomplete retrieval of identified research, reporting bias). | 10-12 |
| Conclusions | 26 | Provide a general interpretation of the results in the context of other evidence, and implications for future research. | 13 |
| **FUNDING** | | | 13 |
| Funding | 27 | Describe sources of funding for the systematic review and other support (e.g., supply of data); role of funders for the systematic review. | 13 |

*From:*  Moher D, Liberati A, Tetzlaff J, Altman DG, The PRISMA Group (2009). Preferred Reporting Items for Systematic Reviews and Meta-Analyses: The PRISMA Statement. PLoS Med 6(6): e1000097. doi:10.1371/journal.pmed1000097

For more information, visit: **www.prisma-statement.org**.

- 1. Supplementary table 2. Characteristics of included studies

| **Authors** | **Year** | **Country** | **Ethnicity** | **Study design** | **Diagnostic technique** | **Type DM** | **Virus** | **N Case** | **N Case+** | **N Control** | **N Control+** | **Age**  **Case*** | **Age Control*** | **Sex case (M/F)** | **Sex control**  **(M/F)** |
| --- | --- | --- | --- | --- | --- | --- | --- | --- | --- | --- | --- | --- | --- | --- | --- |
| Adegoke et al | 2008 | Nigeria | Africans | CC | ELISA | T2DM | HCV | 115 | 1 | 2013 | 45 | 55.4 (9) | 36(5) | 60/55 |  |
| Al Humayed et al | 2018 | Saudi Arabia | Asians | CC | ELISA, RT-PCR | T2DM | HCV | 150 | 12 | 150 | 3 | 55.9(13,4) |  | 187/113 |  |
| Ali et al | 2012 | Ethiopia | Africans | CC | Rapid screening test | T2DM | HCV | 304 | 30 | 300 | 10 | 44.3(15,3) | 26.87(8,3) | 188/116 | 170/130 |
| Balogun et al | 2006 | Nigeria | Africans | CC | Rapid Screening test | T2DM | HCV | 90 | 0 | 90 | 1 | 58.7(10,6) | 59.6(9,9) | 45/45 | 47/43 |
| Cadranel et al | 2008 | France | Europa | CC | ELISA, Immunoblot, PCR, Inolipa | T2DM | HCV | 1166 | 23 | 31750 | 25 | 59.3(2,61) |  | 768/793 |  |
| Chehadeh et al | 2011 | Egypt | Africans | CC | ELISA, nexted-RT-PCR | T2DM | HCV | 438 | 31 | 440 | 4 | 47 | 47 | 278/160 | 255/185 |
| Chen et al | 2006 | Taiwan | Asians | CC | ELISA | T2DM | HCV | 820 | 56 | 905 | 23 | 56.95(11,33) | 53.65(15,28) | 428/392 | 503/402 |
| Costa et al | 2008 | Brazil | Americans | CC | ELISA,PCR, Immunoblot assay | T2DM | HCV | 206 | 3 | 206 | 2 | 55 | 54.6 | 58/148 |  |
| Cuadros et al | 2015 | Egypt | Africans | CC | ELISA,CIA,RT-qPCR | T2DM | HCV | 351 | 74 | 9792 | 904 | 48 | 32 | 136/215 | 4624/5168 |
| Farshadpour et al | 2018 | Iran | Asians | CC | ELISA, RT-PCR | T2DM | HCV | 756 | 11 | 733 | 4 | 54.4(11,6) |  | 203/353 | 257/476 |
| Gebrekristos et al | 2018 | Ethiopia | Africans | CC | Rapid screening test, ELISA | T2DM | HCV | 230 | 64 | 230 | 13 |  |  |  |  |
| Gisi et al | 2017 | Turkey | Asians | CC | Architect Reagent Kit | T2DM | HCV | 1149 | 26 | 1482 | 7 | 56.11(12,71) | 46.93(5,8) | 472/791 | 1464/18 |
| Gulcan et al | 2008 | Turkey | Asians | CC | ELISA, PCR | T2DM | HCV | 617 | 19 | 314 | 4 | 57.46(11,27) | 52.04(10,13) | 224/393 | 105/199 |
| Guney et al | 2005 | Turkey | Asians | CC | PCR | T2DM | HCV | 60 | 5 | 45 | 0 | 54.7(12,8) | 36.7(9,1) | 27/33 | 26/19 |
| Huang et al | 2007 | Taiwan | Asians | CC | ELISA,RT-PCR | T2DM | HCV | 1237 | 96 | 8695 | 546 | 57.1(6,15) | 54.9(5,87) | 640/597 | 3652/5043 |
| Jadoon et al | 2010 | Pakistan | Asians | CC | Rapid screening test, ELISA | T2DM | HCV | 3000 | 410 | 10000 | 496 | 48.19(10,32) | 27 | 1330/1670 |  |
| Jain et al | 2007 | USA | Americans | CC | ELISA, PCR | T2DM | HCV | 163 | 55 | 1366 | 363 | 40 |  |  |  |
| Juttada et al | 2019 | India | Asians | CC | RT-PCR | T2DM | HCV | 258 | 10 | 130 | 1 | 58(10,9) | 48(13,2) |  |  |
| Kaabia et al | 2009 | Tunisia | Africans | CC | EIA | T2DM | HCV | 1269 | 17 | 1315 | 8 | 55.6 | 46.9 | 452/817 | 425/890 |
| Kanwal | 2016 | Paskitan | Asians | CC | ELISA | T2DM | HCV | 100 | 15 | 100 | 3 | 45,66(10,94) |  |  |  |
| Kombi et al | 2018 | DRC | Africans | CC | Rapid screening test, ELISA | T2DM | HCV | 149 | 37 | 5259 | 101 |  |  | 86/63 |  |
| Korkmaz et al | 2015 | Turkey | Asians | CC | Chemilluminescence, Real-time PCR | T2DM | HCV | 727 | 24 | 505 | 7 | 54,10(9,96) | 49,63(11,0) | 206/521 | 155/350 |
| Liu et al | 2019 | China | Asians | CC | ELISA | T2DM | HCV | 2468 | 85 | 3435 | 52 | 59,81(9,21) | 56,38(9,99) | 810/1658 | 1092/2343 |
| Madny et al | 2014 | Sudan | Africans | CC | ELISA | T2DM | HCV | 180 | 3 | 180 | 0 | 44,8 | 38,2 | 79/101 | 150/30 |
| Mason et al | 1999 | USA | Americans | CC | EIA | T2DM | HCV | 594 | 25 | 377 | 6 | 60,5 |  | 321/273 |  |
| Million et al | 2019 | Ethiopia | Africans | CC | ELISA | T2DM | HCV | 305 | 23 | 305 | 7 | 37,7 | 36,3 | 172/133 | 182/123 |
| Ndako et al | 2020 | Nigeria | Africans | CC | ELISA | T2DM | HCV | 180 | 24 | 100 | 9 |  |  | 71/109 | 48/52 |
| Nwokediuko et al | 2008 | Nigeria | Africans | CC | ELISA | T2DM | HCV | 191 | 27 | 134 | 5 | 55,87(11,84) | 54,43(11,16) | 80/111 | 69/65 |
| Ocak et al | 2006 | Turkey | Asians | CC | ELISA, RT-PCR | T2DM | HCV | 67 | 14 | 200 | 20 | 56,1(12,8) | 50,8(14,6) |  |  |
| Okan et al | 2002 | Turkey | Asians | CC | ELISA, RT-PCR | T2DM | HCV | 692 | 52 | 1014 | 1 | 51,9 |  | 260/432 |  |
| Olokoba et al | 2010 | Nigeria | Africans | CC | ELISA | T2DM | HCV | 280 | 26 | 595 | 14 | 51,5(12) | 31,3(7,9) | 108/172 | 571/24 |
| Parolin et al | 2006 | Brazil | Americans | CC | ELISA | T2DM | HCV | 145 | 3 | 16720 | 11 | 55,25(11,42) |  | 49/96 |  |
| Picerno et al | 2002 | Italy | Europa | CC | ELISA, Immunoblot | T2DM | HCV | 254 | 6 | 223 | 3 | 51,66 (5,78) | 47,17 (6,14) | 148/106 | 143/80 |
| Putcharoen et al | 2017 | Thailand | Asians | Cohort |  | T2DM | HCV | 123 | 14 | 1625 | 107 | 35,9 (29,2‐42,9) | 31,9 (27,3‐37,3) | 96/27 | 955/670 |
| Rangarajan et al | 2016 | India | Asians | CC | EIA | T2DM | HCV | 500 | 13 | 500 | 6 |  |  |  |  |
| Rao et al | 2011 | USA | Americans | CC | PCR | T2DM | HCV | 1704 | 252 | 6619 | 1140 | 53,5(5,3) | 51,8(5,8) | 1660/44 | 6317/302 |
| Rudoni et al | 1999 |  | Europa | CC |  | T2DM | HCV | 259 | 8 | 14100 | 6 |  |  |  |  |
| Sangiorgio et al | 2000 | Italy | Europa | CC | ELISA, Immunoblot | T2DM | HCV | 1514 | 115 | 1300 | 30 | 63,05(10,48) |  | 648/846 |  |
| Saxena et al | 2003 | Saudi Arabia | Asians | CC | ELISA, Immunoblot | T2DM | HCV | 54 | 31 | 142 | 50 | 52,8(18,6) | 49,6(18,3) | 32/22 | 67/75 |
| Simo et al | 1996 | Spain | Europa | Cohort | ELISA | T2DM | HCV | 116 | 14 | 6172 | 156 | 59,2 (13,5) | 43,8 (14,6) | 45/71 | 2864/3308 |
| Suliman et al | 2004 | Pakistan | Asians | CC | ELISA | T2DM | HCV | 100 | 3 | 100 | 0 |  |  | 50/50 | 50/50 |
| Wang et al | 2003 | China | Asians | CC | EIA | T2DM | HCV | 211 | 66 | 1533 | 297 |  |  |  |  |
| Yang et al | 2003 | China | Asians | CC | ELISA | T2DM | HCV | 160 | 5 | 223 | 0 |  |  |  |  |
| Caselli et al | 2014 | Italy | Europa | CC | PCR | T2DM | HHV8 | 168 | 96 | 108 | 19 | 65(12) | 44,4(12) | 88/80 | 74/34 |
| Cikomola et al | 2016 | DRC | Africans | CC | ELISA | T2DM | HHV8 | 210 | 180 | 125 | 79 | 60(52-68) | 50(43-55) | 77/133 | 72/53 |
| Cui et al | 2019 | China | Asians | CC | Immunofluorescence assay, RT-PCR | T2DM | HHV8 | 324 | 159 | 376 | 89 | 53(41-64) | 49(37-62) | 193/131 | 171/205 |
| Incani et al | 2020 | Italy | Europa | CC | Immunofluorescence assay, PCR | T2DM | HHV8 | 31 | 15 | 23 | 9 |  |  |  |  |
| Ingianni et al | 2007 | Italy | Europa | CC | Nexted PCR, Southern blot | T2DM | HHV8 | 114 | 27 | 108 | 13 |  |  | 53/61 | 83/23 |
| Piras et al | 2017 | Italy | Europa | CC | PCR | T2DM | HHV8 | 646 | 374 | 363 | 98 |  |  | 360/286 | 187/148 |
| Sobngwi et al | 2008 | France | Africans | CC | Immunofluorescence , ELISA, PCR | T2DM | HHV8 | 106 | 16 | 90 | 36 | 50(10) |  | 70/36 |  |
| Sobngwi et al | 2008 | France | Africans | CC | Immunofluorescence , ELISA, PCR | KPD | HHV8 | 81 | 71 | 90 | 36 | 48(11) |  | 67/14 |  |
| Chen et al | 2006 | China | Asians | CC | ELISA | T2DM | HBV | 820 | 111 | 905 | 112 | 56,95(11,33) | 53,65(15,28) | 428/392 | 503/402 |
| Colloredo et al | 1986 | Italy | Europa | CC | RIA | T2DM | HBV | 322 | 33 | 265 | 23 | 54(10,4) | 54(10,4) | 218/176 |  |
| Demir | 2008 | Turkey | Asians | CC | Real-Time PCR | T2DM | HBV | 100 | 11 | 100 | 3 | 48,7(9,8) | 46,4(13,7) | 51/49 | 42/58 |
| Feirera et al | 2018 | UK | Europa | Cohort |  | T2DM | HBV | 154084 | 477 | 4679010 | 845 | 54,4 | 32 | 91953/68807 | 2345824/2333186 |
| Gisi et al | 2017 | Turkey | Asians | CC | Architect Reagent Kit | T2DM | HBV | 1149 | 46 | 1482 | 16 | 56,11(12,71) | 46,93(5,8) | 472/791 | 1464/18 |
| Gulcan et al | 2008 | Turkey | Asians | CC | ELISA, PCR | T2DM | HBV | 618 | 31 | 314 | 12 | 57,46(11,27) | 52,04(10,13) | 224/393 | 105/199 |
| Guney et al | 2005 | Turkey | Asians | CC | PCR | T2DM | HBV | 60 | 3 | 45 | 2 | 54,7(12,8) | 36,7(9,1) | 27/33 | 26/19 |
| Huang et al | 2007 | Taiwan | Asians | CC | ELISA | T2DM | HBV | 1237 | 155 | 8695 | 1208 | 57,1(6,15) | 54,9(5,87) | 640/597 | 3652/5043 |
| Juttada et al | 2019 | India | Asians | CC | RT-PCR | T2DM | HBV | 258 | 35 | 130 | 1 | 58(10,9) | 48(13,2) |  |  |
| Kombi et al | 2018 | DRC | Africans | CC | Rapid screening test, ELISA | T2DM | HBV | 149 | 4 | 5259 | 186 |  |  | 86/63 |  |
| Korkmaz et al | 2015 | Turkey | Asians | CC | Chemilluminescence, Real-time PCR | T2DM | HBV | 727 | 28 | 505 | 15 | 54,10(9,96) | 49,63(11,0) | 206/521 | 155/350 |
| Liu et al | 2019 | China | Asians | CC | ELISA | T2DM | HBV | 2468 | 135 | 3435 | 183 | 59,81(9,21) | 56,38(9,99) | 810/1658 | 1092/2343 |
| Lu et al | 2017 | China | Asians | CC | ELISA, Chemilluminescence | T2DM | HBV | 1365 | 184 | 1365 | 137 | 54(45-63) | 54(45-63) | 847/518 | 847/518 |
| Mekonnen et al | 2014 | Ethiopia | Africans | CC | Rapid screening test | T2DM | HBV | 108 | 4 | 108 | 4 | 37,6(13) | 29,2(10.4) | 57/51 | 63/45 |
| Million et al | 2019 | Ethiopia | Africans | CC | ELISA | T2DM | HBV | 305 | 26 | 305 | 14 | 37,7 | 36,3 | 172/133 | 182/123 |
| Ndako et al | 2020 | Nigeria | Africans | CC | ELISA | T2DM | HBV | 180 | 24 | 100 | 9 |  |  | 71/109 | 48/52 |
| OKan et al | 2002 | Turkey | Asians | CC | ELISA, PCR | T2DM | HBV | 692 | 37 | 1014 | 52 | 51,9 |  | 260/432 | 881/133 |
| Onyekwere et al | 2002 | Nigeria | Africans | CC |  | T2DM | HBV | 100 | 20 | 80 | 14 |  |  |  |  |
| Putcharoen et al | 2017 | Thailand | Asians | Cohort |  | T2DM | HBV | 123 | 6 | 1625 | 136 | 35,9 (29,2‐42,9) | 31,9 (27,3‐37,3) | 96/27 | 955/670 |
| Rangarajan et al | 2016 | India | Asians | CC | EIA | T2DM | HBV | 500 | 42 | 500 | 39 |  |  |  |  |
| Sangiorgio et al | 2000 | Italy | Europa | CC | ELISA | T2DM | HBV | 1514 | 107 | 1300 | 21 | 63,05(10,49) |  | 648/846 |  |
| Shen et al | 2016 | China | Asians | CC | ELISA, RT-PCR | T2DM | HBV | 207 | 95 | 207 | 66 | 51,34(10,87) | 50,16(10,49) | 163/44 | 163/44 |
| Suliman et al | 2004 | pakistan | Asians | CC | ELISA | T2DM | HBV | 100 | 4 | 100 | 5 |  |  | 50/50 | 50/50 |
| Wang et al | 2003 | China | Asians | CC | ELISA | T2DM | HBV | 211 | 27 | 1533 | 193 |  |  |  |  |
| Yang et al | 2003 | China | Asians | CC | ELISA | T2DM | HBV | 160 | 7 | 223 | 12 |  |  |  |  |
| Zhang X et al | 2019 | China | Asians | Cohort | ELISA | T2DM | HBV | 4982 | 265 | 182710 | 7038 | 62,8(9,8) | 47,3(17,7) | 1944/3038 | 77328/105382 |
| Zhu et al | 2016 | China | Asians | CC | ELISA, PCR | T2DM | HBV | 338 | 72 | 3039 | 472 |  |  | 185/153 | 1405/1634 |
| Haq et al | 2017 | Canada | Americans | CC | ELISA | T2DM | CMV | 30 | 16 | 40 | 28 | 72,35 (3,8) | 73,37 (4,94) | 21/9 | 24/16 |
| Lohr et al | 1990 | Belgium | Europa | CC | PCR, hybridisation assay | T2DM | CMV | 32 | 14 | 50 | 1 | 69(7) | 63,2(14) | 16/16 | 27/23 |
| Lohr et al | 1992 | Belgium | Europa | CC | PCR, hybridisation assay | T2DM | CMV | 19 | 10 | 15 | 0 | 69,6(10,1) | 65,0(16,9) | 11/8 | 9/6 |
| Roberts et al | 2005 | USA | Americans | CC | Indirect fluoresecent immunoassay | T2DM | CMV | 83 | 81 | 30 | 26 | 62 | 49 |  |  |
| Roubalova et al | 2007 | Czech Republic | Europa | CC | ELISA, nexted PCR | T2DM | CMV | 44 | 34 | 44 | 39 | 59 |  | 24/20 |  |
| Sun et al | 2005 | China | Asians | CC | ELISA | T2DM | HSV1 | 206 | 95 | 1360 | 494 | 46,1 | 36,3 | 158/48 | 1111/249 |
| Guney et al | 2005 | Turkey | Asians | CC | ELISA | T2DM | TTV | 60 | 16 | 45 | 5 | 54,7(12,8) | 36,7(9,1) | 27/33 | 26/19 |
| Roberts et al | 2005 | USA | Americans | CC | Indirect fluoresecent immunoassay | T2DM | Parvovirus B19 | 83 | 77 | 30 | 25 | 62 | 49 |  |  |
| Roberts et al | 2005 | USA | Americans | CC | Indirect fluoresecent immunoassay | T2DM | Coxackie B virus | 83 | 41 | 30 | 18 | 62 | 49 |  |  |
| Guney et al | 2005 | Turkey | Asians | CC | ELISA | T2DM | HGV | 60 | 0 | 45 | 1 | 54,7(12,8) | 36,7(9,1) | 27/33 | 26/19 |
| McGurnaghan et al | 2021 | UK | Europa | Cohort | PCR | T2DM | SARS-CoV-2 | 319349 | 2724 | 5143951 | 4081 | 66,7(56,3-75,8) | | 180486/138863 |  |
| Ruiz et al | 2021 | Norway | Europa | Cohort |  | T2DM | H1N1 Virus | 149432 | 126 | 2842796 | 1163 | 65,2 | 53,2 | 80500/68932 | 1391511/1451285 |
| *Mean (SD)/Median (IQR) ; CC: Case Control, DM: Diabetes mellitus; T2DM: Type 2 Diabetes mellitus; HCV: Hepatitis C Virus; HHV8: Human Herpes Virus 8 ; HBV: Hepatitis B Virus; CMV: Cytomegalovirus ; HSV1: Herpes Simplex Virus 1 ; TTV: Tranfusion ransmitted Virus ; HGV: Hepatitis G Virus; SARS-CoV-2 : Severe Acute Respiratory Syndrome Coronavirus 2; KPD: Ketosis-Prone Diabetes; DRC: Democratic Republic of the Congo | | | | | | | | | | | | | | | |

- 1. Supplementary table 3. Quality assessment of included studies

| **Authors** | **Year** | **Virus** | **Selection** | **Comparability** | **Exposure** | **Outcome** | **Final score** | **Risk of bias** |
| --- | --- | --- | --- | --- | --- | --- | --- | --- |
| Adegoke et al | 2008 | HCV | 3 | 1 | 2 |  | 6 | Moderate |
| Al Humayed et al | 2018 | HCV | 4 | 2 | 2 |  | 8 | Low |
| Ali et al | 2012 | HCV | 4 | 1 | 2 |  | 7 | Low |
| Balogun et al | 2006 | HCV | 3 | 2 | 2 |  | 7 | Low |
| Cadranel et al | 2008 | HCV | 4 | 1 | 3 |  | 8 | Low |
| Chehadeh et al | 2011 | HCV | 3 | 1 | 3 |  | 7 | Low |
| Chen et al | 2006 | HCV | 4 | 1 | 3 |  | 8 | Low |
| Costa et al | 2008 | HCV | 2 | 1 | 2 |  | 5 | Moderate |
| Cuadros et al | 2015 | HCV | 2 | 1 | 2 |  | 5 | Moderate |
| Farshadpour et al | 2018 | HCV | 4 | 1 | 2 |  | 7 | Low |
| Gebrekristos et al | 2018 | HCV | 3 | 1 | 3 |  | 7 | Low |
| Gisi et al | 2017 | HCV | 3 | 1 | 2 |  | 6 | Moderate |
| Gulcan et al | 2008 | HCV | 4 | 1 | 2 |  | 7 | Low |
| Guney et al | 2005 | HCV | 4 | 1 | 2 |  | 7 | Low |
| Huang et al | 2007 | HCV | 3 | 1 | 3 |  | 7 | Low |
| Jadoon et al | 2010 | HCV | 4 | 1 | 3 |  | 8 | Low |
| Jain et al | 2007 | HCV | 2 | 1 | 3 |  | 6 | moderate |
| Juttada et al | 2019 | HCV | 4 | 1 | 3 |  | 8 | Low |
| Kaabia et al | 2009 | HCV | 4 | 1 | 2 |  | 7 | Low |
| Kanwal | 2016 | HCV | 3 | 1 | 3 |  | 7 | Low |
| Kombi et al | 2018 | HCV | 4 | 1 | 3 |  | 8 | Low |
| Korkmaz et al | 2015 | HCV | 4 | 1 | 3 |  | 8 | Low |
| Liu et al | 2019 | HCV | 3 | 1 | 3 |  | 7 | Low |
| Madny et al | 2014 | HCV | 3 | 1 | 2 |  | 6 | moderate |
| Mason et al | 1999 | HCV | 4 | 1 | 2 |  | 7 | Low |
| Million et al | 2019 | HCV | 4 | 1 | 3 |  | 8 | Low |
| Ndako et al | 2020 | HCV | 3 |  | 2 |  | 5 | Moderate |
| Nwokediuko et al | 2008 | HCV | 3 | 1 | 2 |  | 6 | Moderate |
| Ocak et al | 2006 | HCV | 2 | 1 | 2 |  | 5 | Moderate |
| Okan et al | 2002 | HCV | 4 | 1 | 2 |  | 7 | Low |
| Olokoba et al | 2010 | HCV | 4 | 1 | 3 |  | 8 | Low |
| Parolin et al | 2006 | HCV | 4 | 1 | 3 |  | 8 | Low |
| Picerno et al | 2002 | HCV | 4 | 1 | 2 |  | 7 | Low |
| Putcharoen et al | 2017 | HCV | 3 | 1 |  | 2 | 6 | Moderate |
| Rangarajan et al | 2016 | HCV | 4 | 1 | 3 |  | 8 | Low |
| Rao et al | 2011 | HCV | 3 | 1 | 3 |  | 7 | Low |
| Rudoni et al | 1999 | HCV | 3 | 1 | 3 |  | 7 | Low |
| Sangiorgio et al | 2000 | HCV | 3 | 1 | 3 |  | 7 | Low |
| Saxena et al | 2003 | HCV | 3 | 1 |  | 2 | 6 | Moderate |
| Simo et al | 1996 | HCV | 4 | 1 | 2 |  | 7 | Low |
| Suliman et al | 2004 | HCV | 2 | 1 | 2 |  | 5 | Moderate |
| Wang et al | 2003 | HCV | 2 | 1 | 3 |  | 6 | Moderate |
| Yang et al | 2003 | HCV | 3 | 1 | 3 |  | 7 | Low |
| Caselli et al | 2014 | HHV8 | 4 | 1 | 2 |  | 7 | Low |
| Cikomola et al | 2016 | HHV8 | 3 | 1 | 2 |  | 6 | Moderate |
| Cui et al | 2019 | HHV8 | 4 | 1 | 3 |  | 8 | Low |
| Incani et al | 2020 | HHV8 | 2 | 1 | 2 |  | 5 | Moderate |
| Ingianni et al | 2007 | HHV8 | 4 | 1 | 3 |  | 8 | Low |
| Piras et al | 2017 | HHV8 | 4 | 1 | 3 |  | 8 | Low |
| Sobngwi et al | 2008 | HHV8 | 4 | 1 | 2 |  | 7 | Low |
| Sobngwi et al | 2008 | HHV8 | 4 | 1 | 2 |  | 7 | Low |
| Chen et al | 2006 | HBV | 4 | 1 | 3 |  | 8 | Low |
| Colloredo et al | 1986 | HBV | 3 | 1 | 2 |  | 6 | Moderate |
| Demir | 2008 | HBV | 3 | 2 | 2 |  | 7 | Low |
| Feirera et al | 2018 | HBV | 4 | 1 |  | 3 | 8 | Low |
| Gisi et al | 2017 | HBV | 3 | 1 | 2 |  | 6 | Moderate |
| Gulcan et al | 2008 | HBV | 4 | 1 | 2 |  | 7 | Low |
| Guney et al | 2005 | HBV | 4 | 1 | 2 |  | 7 | Low |
| Huang et al | 2007 | HBV | 3 | 1 | 3 |  | 7 | Low |
| Juttada et al | 2019 | HBV | 4 | 1 | 3 |  | 8 | Low |
| Kombi et al | 2018 | HBV | 4 | 1 | 3 |  | 8 | Low |
| Korkmaz et al | 2015 | HBV | 4 | 1 | 3 |  | 8 | Low |
| Liu et al | 2019 | HBV | 3 | 1 | 3 |  | 7 | Low |
| Lu et al | 2017 | HBV | 3 | 1 | 3 |  | 7 | Low |
| Mekonnen et al | 2014 | HBV | 3 | 1 | 2 |  | 6 | Moderate |
| Million et al | 2019 | HBV | 4 | 1 | 3 |  | 8 | Low |
| Ndako et al | 2020 | HBV | 3 |  | 2 |  | 5 | moderate |
| OKan et al | 2002 | HBV | 4 | 1 | 2 |  | 7 | Low |
| Onyekwere et al | 2002 | HBV | 3 | 1 | 3 |  | 7 | Low |
| Putcharoen et al | 2017 | HBV | 3 | 1 |  | 2 | 6 | moderate |
| Rangarajan et al | 2016 | HBV | 4 | 1 | 3 |  | 8 | Low |
| Sangiorgio et al | 2000 | HBV | 3 | 1 | 3 |  | 7 | Low |
| Shen et al | 2016 | HBV | 4 | 1 | 3 |  | 8 | Low |
| Suliman et al | 2004 | HBV | 2 | 1 | 2 |  | 5 | Moderate |
| Wang et al | 2003 | HBV | 2 | 1 | 3 |  | 6 | Moderate |
| Yang et al | 2003 | HBV | 3 | 1 | 3 |  | 7 | Low |
| Zhang X et al | 2019 | HBV | 4 | 1 |  | 3 | 8 | Low |
| Zhu et al | 2016 | HBV | 4 | 1 | 2 |  | 7 | Low |
| Haq et al | 2017 | CMV | 4 | 1 | 3 |  | 8 | Low |
| Lohr et al | 1990 | CMV | 3 | 1 | 3 |  | 7 | Low |
| Lohr et al | 1992 | CMV | 3 | 1 | 3 |  | 7 | Low |
| Roberts et al | 2005 | CMV | 3 | 1 | 2 |  | 6 | Moderate |
| Roubalova et al | 2007 | CMV | 3 | 1 | 2 |  | 6 | Moderate |
| Sun et al | 2005 | HSV1 | 2 | 1 | 2 |  | 5 | Moderate |
| Guney et al | 2005 | TTV | 4 | 1 | 2 |  | 7 | Low |
| Roberts et al | 2005 | Parvovirus B19 | 3 | 1 | 2 |  | 6 | Moderate |
| Roberts et al | 2005 | Coxackie B virus | 3 | 1 | 2 |  | 6 | Moderate |
| Guney et al | 2005 | HGV | 4 | 1 | 2 |  | 7 | Moderate |
| McGurnaghan et al | 2021 | SARS-CoV-2 | 4 | 1 |  | 3 | 8 | Low |
| Ruiz et al | 2021 | H1N1 Virus | 4 | 1 |  | 3 | 8 | Low |

2.4. Supplementary table 4. Subgroup analyses of the association between non-autoimmune diabetes mellitus and type specific virus

|  | **OR (95%CI)** | **95% Prediction interval** | **N Studies** | **N Cases** | **N controls** | **H (95%CI)** | **I² (95%CI)** | **P heterogeneity** | **P. Egger test** | **P.subgroup difference** |
| --- | --- | --- | --- | --- | --- | --- | --- | --- | --- | --- |
| **CMV** |  |  |  |  |  |  |  |  |  |  |
| **Ethnicity** |  |  |  |  |  |  |  |  |  | 0,334 |
| Americans | 1.4 [0.3-7.7] | NA | 2 | 113 | 70 | 2.5 [1.2-5] | 83.7 [32.6-96.1] | NA | NA |  |
| Europa | 6.3 [0.5-80.7] | [0->1000] | 3 | 95 | 109 | 3 [1.8-4.9] | 88.6 [68.7-95.9] | NA | 0,682 |  |
| **Diagnostic techniques** |  |  |  |  |  |  |  |  |  | < 0.001 |
| Immunoassay | 1.4 [0.3-7.7] | NA | 2 | 113 | 70 | 2.5 [1.2-5] | 83.7 [32.6-96.1] | NA | NA |  |
| Immunoassay+ Molecular assay | 0.4 [0.1-1.4] | NA | 1 | 44 | 44 | NA | NA | NA | NA |  |
| Molecular assay | 36.8 [6.6-203.4] | NA | 2 | 51 | 65 | 1 | 0 | NA | NA |  |
| **HBV** |  |  |  |  |  |  |  |  |  |  |
| **Ethnicity** |  |  |  |  |  |  |  |  |  | 0,116 |
| Africans | 1.4 [0.9-2] | [0.7-2.5] | 5 | 842 | 5852 | 1 [1-1.8] | 0 [0-70.6] | NA | 0,261 |  |
| Asians | 1.3 [1.1-1.5] | [0.7-2.2] | 19 | 16115 | 207927 | 1.8 [1.4-2.3] | 68.9 [50.1-80.6] | NA | 0,502 |  |
| Europa | 4.7 [1.4-16.2] | [0->1000] | 3 | 155920 | 4680575 | 7.3 [5.4-9.9] | 98.1 [96.6-99] | NA | 0,514 |  |
| **Study design** |  |  |  |  |  |  |  |  |  | 0,499 |
| Case control | 1.4 [1.2-1.7] | [0.6-3.1] | 24 | 13688 | 31009 | 1.9 [1.6-2.3] | 72.5 [58.7-81.7] | NA | 0,16 |  |
| Cohort | 2.5 [0.5-12.7] | [0->1000] | 3 | 159189 | 4863345 | 20.9 [18-24.3] | 99.8 [99.7-99.8] | NA | 0,715 |  |
| **Diagnostic techniques** |  |  |  |  |  |  |  |  |  | 0,139 |
| Immunoassay | 1.3 [1.1-1.7] | [0.6-3.1] | 18 | 16362 | 209379 | 2.1 [1.6-2.6] | 76.3 [62.7-84.9] | NA | 0,793 |  |
| Immunoassay + Molecular assay | 1.5 [1.2-1.9] | [1-2.4] | 4 | 1890 | 4065 | 1 [1-1.6] | 0 [0-60.2] | NA | 0,411 |  |
| Molecular assay | 4.1 [1.5-11.3] | [0-7661] | 3 | 418 | 275 | 1.5 [1-2.8] | 54 [0-86.8] | NA | 0,113 |  |
| **HCV** |  |  |  |  |  |  |  |  |  |  |
| **Ethnicity** |  |  |  |  |  |  |  |  |  | 0,087 |
| Africans | 3.8 [2.4-5.9] | [0.8-17.4] | 13 | 4082 | 20753 | 2.5 [2-3.2] | 83.8 [73.7-90] | NA | 0,761 |  |
| Americans | 2.5 [0.9-7.3] | [0-131.7] | 5 | 2812 | 25288 | 3.2 [2.3-4.6] | 90.5 [80.7-95.3] | NA | 0,098 |  |
| Asians | 2.5 [2-3.2] | [1.3-5] | 20 | 13249 | 31831 | 1.9 [1.5-2.4] | 71.7 [55.7-82] | NA | 0,705 |  |
| Europa | 9.2 [3-28.9] | [0.1-693.2] | 5 | 3309 | 53545 | 3.7 [2.7-5.1] | 92.7 [85.9-96.2] | NA | 0,1 |  |
| **Study design** |  |  |  |  |  |  |  |  |  | 0,002 |
| Case control | 3.9 [2.9-5.4] | [0.7-21.8] | 40 | 21571 | 123031 | 2.8 [2.4-3.1] | 86.8 [83-89.8] | NA | 0,016 |  |
| Cohort | 1.4 [0.8-2.5] | [0-995.3] | 3 | 1881 | 8386 | 2.8 [1.7-4.8] | 87.5 [64.8-95.6] | NA | 0,108 |  |
| **Diagnostic techniques** |  |  |  |  |  |  |  |  |  | < 0.001 |
| Immunoassay | 3.6 [2.7-4.7] | [1.1-11.5] | 26 | 14478 | 53733 | 2.1 [1.8-2.5] | 77.5 [67.5-84.5] | NA | 0,255 |  |
| Immunoassay + Molecular assay | 3.6 [2-6.6] | [0.4-32.9] | 12 | 6570 | 55165 | 3.3 [2.7-4.1] | 91.1 [86.3-94.2] | NA | 0,077 |  |
| Molecular assay | 0.8 [0.7-1] | [0.3-2.2] | 3 | 2022 | 6794 | 1.7 [1-3.1] | 63.8 [0-89.6] | NA | 0,012 |  |
| **HHV8** |  |  |  |  |  |  |  |  |  |  |
| **Ethnicity** |  |  |  |  |  |  |  |  |  | 0,61 |
| Africans | 2.1 [0.4-12.1] | [0->1000] | 3 | 397 | 305 | 5.3 [3.6-7.6] | 96.4 [92.4-98.3] | NA | 0,712 |  |
| Asians | 3.1 [2.3-4.3] | NA | 1 | 324 | 376 | NA | NA | NA | NA |  |
| Europa | 3.7 [2.9-4.6] | [2.2-6.1] | 4 | 959 | 602 | 1.6 [1-2.8] | 60.8 [0-86.9] | NA | 0,476 |  |
| **Type of DM** |  |  |  |  |  |  |  |  |  | 0,004 |
| KPD | 10.7 [4.9-23.3] | NA | 1 | 81 | 90 | NA | NA | NA | NA |  |
| T2D | 2.2 [1.1-4.5] | [0.2-26.1] | 7 | 1599 | 1193 | 3.2 [2.3-4.2] | 90 [81.9-94.4] | NA | 0,284 |  |
| **Diagnostic techniques** |  |  |  |  |  |  |  |  |  | 0,571 |
| Immunoassay | 3.5 [2.1-5.9] | NA | 1 | 210 | 125 | NA | NA | NA | NA |  |
| Immunoassay + Molecular assay | 1.9 [0.5-7.1] | [0-996.3] | 4 | 542 | 579 | 4.4 [3.1-6.2] | 94.8 [89.6-97.4] | NA | 0,67 |  |
| Molecular assay | 3.8 [3-4.9] | [0.8-18] | 3 | 928 | 579 | 1.5 [1-2.9] | 58.1 [0-88.1] | NA | 0,906 |  |

1. **Supplementary figures**
   1. Supplementary figure 1. Study selection process


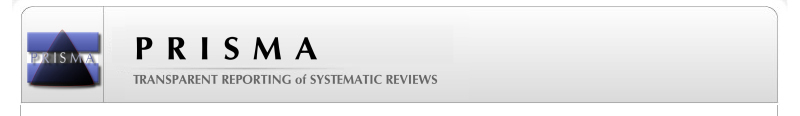
**PRISMA 2009 Flow Diagram**

Studies included in meta-analysis

(association between diabetes and Virus)
(n = 90 studies)

Full-text articles excluded, with reasons
(n = 117)

60 no relevant data

26 no sufficient data for the estimation of OR

8 case report

5 duplicates

3 Letters, editorial

15 reviews

4 not available

Articles included in qualitative synthesis
(n = 68 articles)

Full-text articles assessed for eligibility
(n = 185)

Records excluded
(n = 2716)

Records screened
(n = 2901)

Records after duplicates removed
(n = 2901)

Additional records identified through other sources
(n = 26)

Records identified through database searching
(n = 3110)

## Identification

## Screening

## Eligibility

## Included

*From:*  Moher D, Liberati A, Tetzlaff J, Altman DG, The PRISMA Group (2009). *P*referred *R*eporting *I*tems for *S*ystematic Reviews and *M*eta-*A*nalyses: The PRISMA Statement. PLoS Med 6(7): e1000097. doi:10.1371/journal.pmed1000097

**For more information, visit** [**www.prisma-statement.org**](http://www.consort-statement.org/)**.**

- 1. Supplementary figure 2. Funnel plot for publication bias for HCV infection risk in non-autoimmune DM individuals


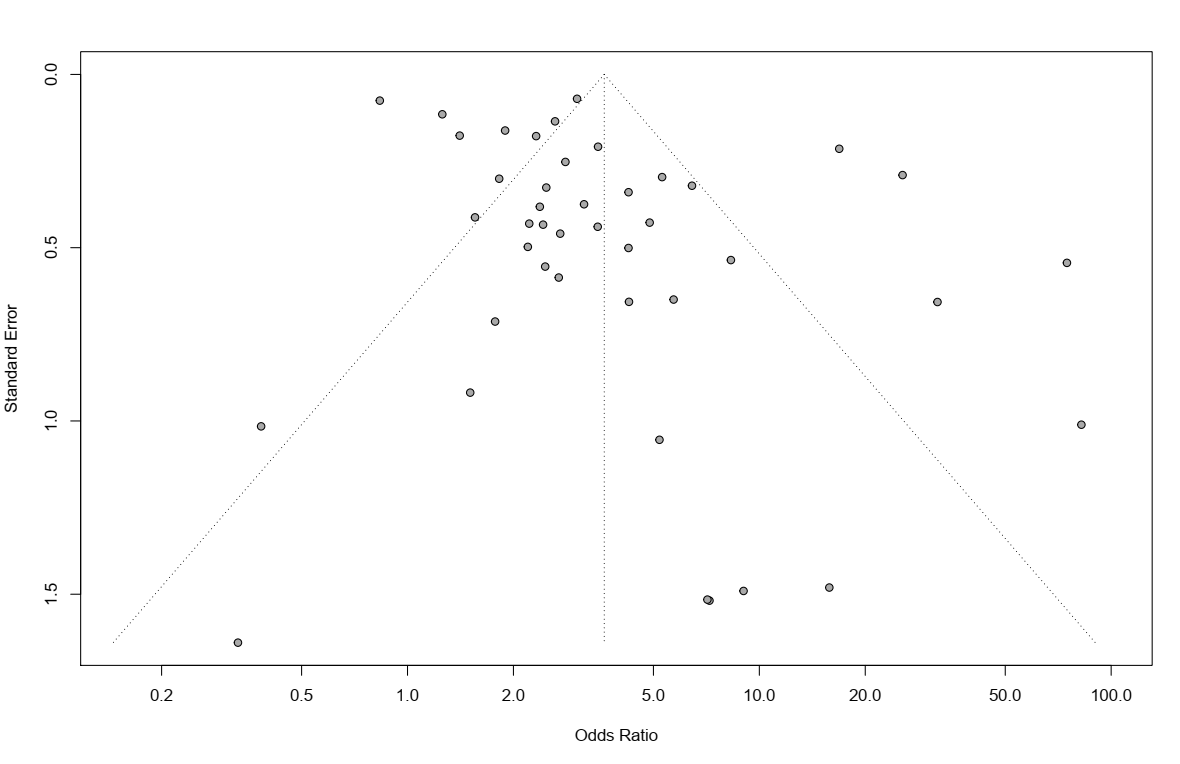


- 1. Supplementary figure 3. Funnel plot for publication bias for HHV8 infection risk in non-autoimmune DM individuals


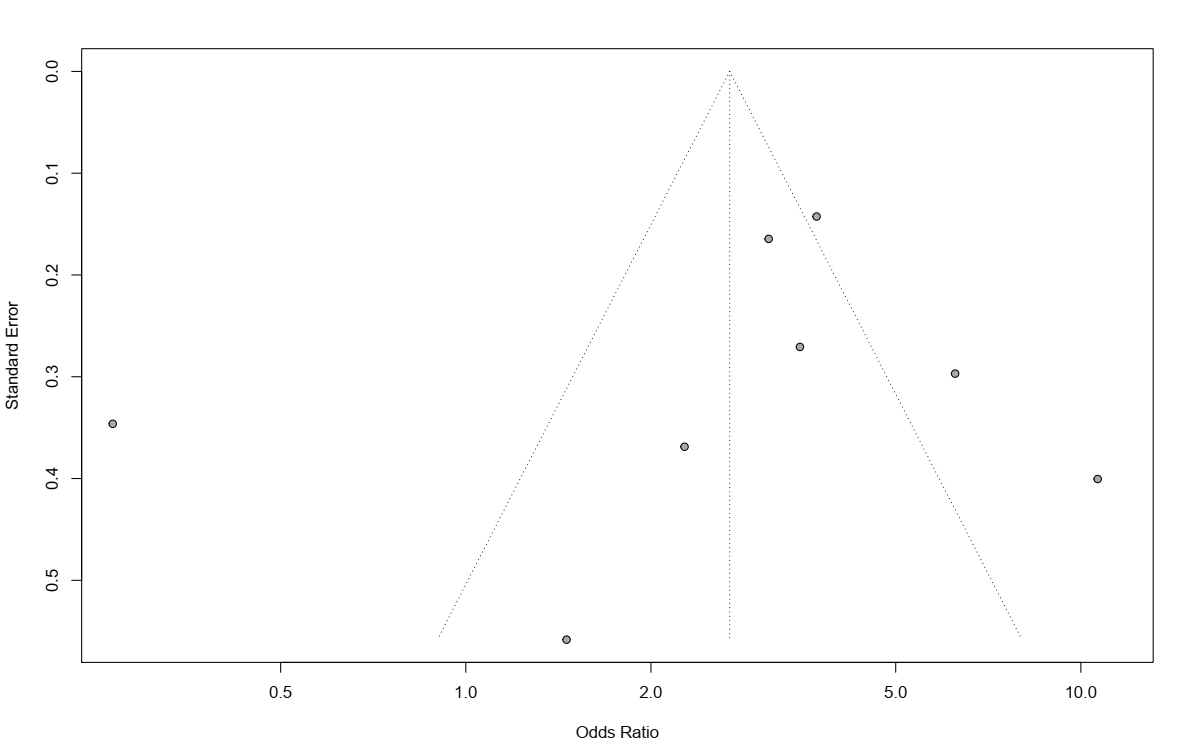


- 1. Supplementary figure 4. Funnel plot for publication bias for HBV infection risk in non-autoimmune DM individuals


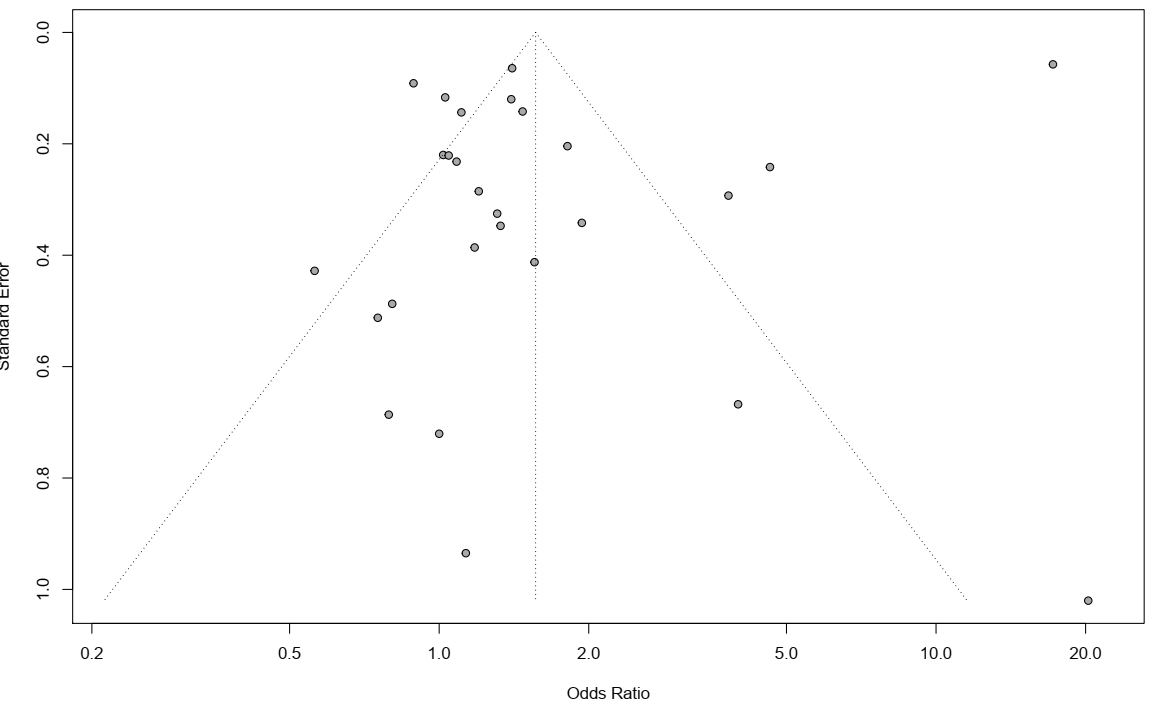


- 1. Supplementary figure 5. Funnel plot for publication bias for CMV infection risk in non-autoimmune DM individuals


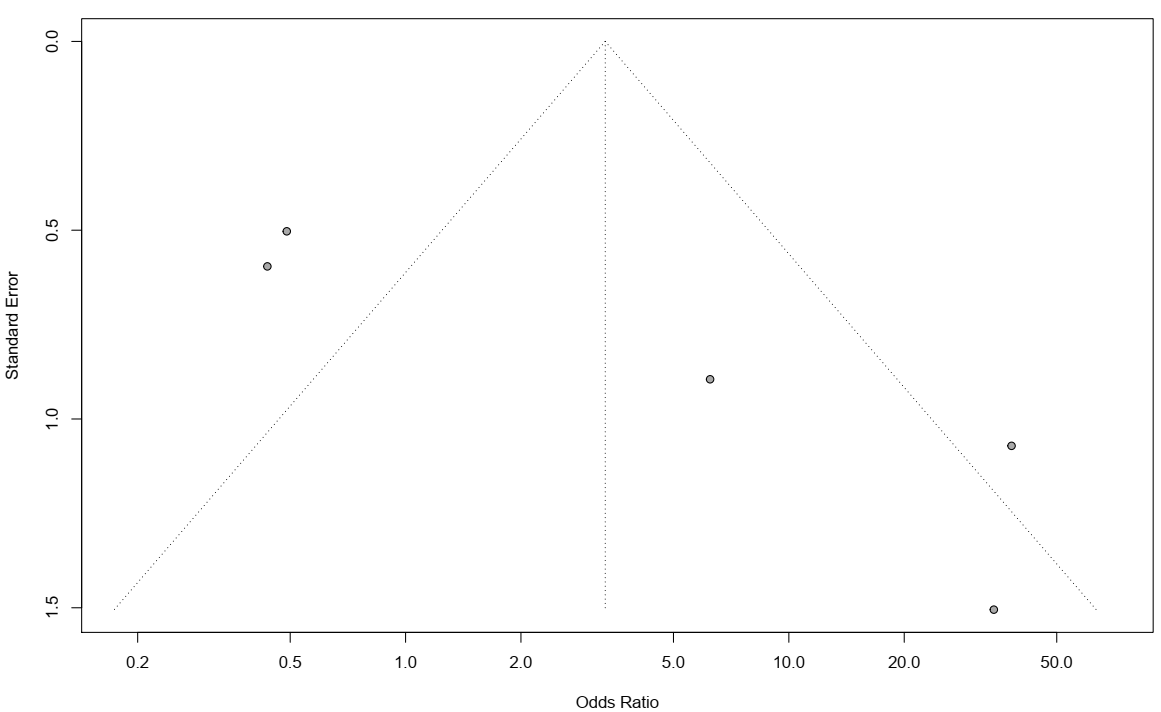


**Supplementary materials figures legends and tables**

Supplementary tables

S Table 1. PRISMA 2009 checklist

S Table 2. Characteristics of included studies

S Table 3. Quality assessment of included studies

S Table 4. Subgroup analyses of the association between non-autoimmune diabetes mellitus and type specific virus

Supplementary figures

S Figure 1. Study selection process

S Figure 2. Funnel plot for publication bias for HCV infection risk in non-autoimmune DM individuals

S Figure 3. Funnel plot for publication bias for HHV8 infection risk in non-autoimmune DM individuals

S Figure 4. Funnel plot for publication bias for HBV infection risk in non-autoimmune DM individuals

S Figure 5. Funnel plot for publication bias for CMV infection risk in non-autoimmune DM individuals
